# Therapeutic Effects of Aconite

**Published:** 1850-05

**Authors:** 


*Therapeutic Effects of Aconite.* By Dr. TEISSIER, of Lyons. Condensed and translated for this Journal, by J. A. S. MILLIGAN, M. D.

We find in the Revue Medico-Chirurgicale an article on this subject by Dr. Teissier, of Lyons. After alluding to the discrepancy in the published opinions of different authors on the therapeutic value of the preparations of aconite, and explaining this discrepancy by a want of similarity of cases in which the remedy was employed, he proceeds to record the results of his researches, undertaken for the purpose of defining in which disease this medicine was indicated.

Dr. T. has examined aconite with reference to its narcotic and antiphlogistic actions—the two modes of action usually ascribed to it. That this remedy possesses narcotic properties, he thinks there can be no doubt. Its power, however, was found to be more feeble than that of opium, belladonna, or the datura, and the field for its applicability more circumscribed. It afforded no relief to patients affected with exostosis, cancer, nephritis, paronychia, etc., but was very efficacious in assuaging the pains of diseases recognizing for their cause a derangement of the functions of the skin, as, for example, rheumatism, angina, odontalgia caused by cold, etc. Dr. T. concludes, therefore, that aconite may be of great service to practitioners as a narcotic, but that this is not its chief property. He has administered aconite in many diseases for the cure of which blood-letting is deemed by all practitioners indispensible, such as peritonitis, apoplexy,and hypertrophy of the heart ; but he has never met with a single case in which he was convinced that this medicine acted as a useful substitute for venesection, or as a directly antiphlogistic remedy. He does not wish to be understood as affirming, however, that anconite never acts as antiphlogistic ; for he will show hereafter, that in certain diseases, as in measles, scarlatina, etc., it may exert a depressive influence on the circulation : he thinks that it produces this effect, however, indirectly, and by regulating another function, the derangement of which had caused the disease.

According to Dr. Teissier the action of aconite is direced chiefly to the skin. Its special property is to eliminate from the cutaneous tissue all noxious principles, and to re-establish the disordered functions of this organ. He recommends the preparations of aconite, therefore, in all diseases caused by a "derangement of the cutaneous secretion, and consecutively by a catarrhal *jetee* on the articulations (articular rheumatism), on the muscles (muscular rheumatism, lumbago), on the nerves, (sciatica, odontalgia,) on the mucous membranes, (coryza, angina, bronchitis, grippe, catarrhal fever fever, etc.)" Dr. T. recommends aconite also at the commencement of all eruptive fevers.

We will notice briefly some of the diseases in which he has found aconite particularly useful.

*Courbature.*—This mild disease, caused by a suppression of perspiration, and characterized by pains in the limbs, shivering, lassitude, headache, and general aching, says Dr. T., may serve as a type for the administra tion of aconite. In this affection he prescribed from 5 to 10 drops of the tincture during the day.

*Catarrhal Fever.*—Dr. T. found aconite equally serviceable in catarrhal fever. The therapeutic indications in this fever are : 1. To re-establish the functions of the skin ; 2. To combat the local irritation of the nose, throat or bronchi. He affirms that aconite fulfils perfectly the former of these indications; but that in carrying out the latter, it requires, the assistance of other means, such as opiates, vesicatories, etc.

*Angina, Acute Bronchitis.*—"With MM. Tessier (of Paris) and Gabalda, we have frequently known aconite," says the writer, "to exert a happy influence in simple angina, and in acute pulmonary catarrah, mitigating readily the pains of deglutition in the former, and quieting the severe cough of the latter. We have found it only necessary in these cases to administer from 5 to 10 drops of the tincture every day."

*Grippe.*—Dr. T. states that in this disease, which bears a striking resemblance to courbature and catarrhal fever, aconite is very serviceable: it restores the healthy action of the skin, relieves the painful sensations of the disease, and abridges its duration. He considers it worthy of remark, too, that those patients who made use of this medicine did not retain so long the feeling of depression and general aching, which so frequently follows an attack of grippe.

*Rheumatism.*—Dr. T. has not found aconite equally useful in all cases of rheumatism. He derived the most decided benefit from it in cases of recent origin, attended neither by swelling nor fever, or in which these symptoms were but slightly developed. In these cases he thinks it preferable to blood-letting, which only combats the pain, which does not remove the rheumatic principle, and which frequently predisposes the part affected to stiffness, swelling, and a recurrence of the evil: to morphine and belladona, because they are not so well tolerated by the stomach, and because they too are merely paliatives. In acute articular rheumatism, with fever and decided tumefaction, the writer has found aconite less effectual than in the cases just mentioned. But even in these cases it may be administered at the commencement, in order to diminish the fluxion to the joints.

In chronic rheumatism, aconite did not prove so serviceable as in cases of recent origin. Dr. Teissier has known instances, however, where obstinate rheumatic pains of several years duration were overcome by the persevering employment of this remedy. He states, further, that aconite is undoubtedly efficacious, when its use is continued for several months, in subduing predispositions to rheumatism. This result it produces by restoring the functions of the skin, especially by promoting the insensible perspiration, and by neutralizing the influence of atmospheric moisture and of the rheumatic principle.

The dose of aconite, the writer observes, must be much larger in rheumatism, than in the diseases previously noticed. We should begin, he says, by giving from 10 to 20 drops of the tincture, and increase the dose gradually to 4, 6, or even 8 grammes a-day.

*Eruptive Fevers.*—Dr. T. has employed this remedy very successfully in exanthematous fevers, in rubeola, variola, scarlatina, urticaria, miliaria, erysipelas, etc. He affirms that in all these diseases, but especially in the measles of children, aconite exerted a happy influence on the fever and on the eruption, which came out with readiness, and ran its course with remarkable regularity. "But," he adds, "if complications occur, it will be necessary to combat them by the other means which experience has sanctioned."

*Erysipelas.*—In this section, the writer treats of the action of aconite in erysipelas complicating wounds. He has known aconite to afford very prompt relief in traumatic erysipelas, attended with fever, rigors, inclination to vomit, temporary delirium, etc. He remembers, particularly, two patients, who were affected very seriously with this disease, but who were relieved, in a manner really surprising, in twenty-four hours. He recommends very highly the employment of this remedy in hospitals where traumatic erysipelas is so common.

*Pneumonia.*—Dr. T. has administered aconite in a great many cases of pneumonia. "On the success which has followed the employment of aconite in this disease," says he, "the homœopathists base their assertion, that this medicine may be usefully substituted for blood-letting." He has found aconite serviceable in the early stages of pneumonia, before the inflammation had become decided ; but when once auscultation had proved that the tissue of the lung was gorged wlth blood and denser than in the normal state, he has found this medicine insufficient, and has been obliged to resort to antimonials. He dees not think, therefore, that it follows as a natural consequence, because aconite contributes to the moderating of the febrile symproms at the commencement of the disease, that it is for this reason an antiphlogistic remedy, and that it acts like venesection. He says, further, that he does not believe blood-letting to be indispensable in the treatment of pneumonia ; that in fact we may do without it in the majority of cases, excepting those very rare instances where the oppression is very great.

The writer remarks, that all he has said respecting pneumonia, is applicable also to pleurisy. "Aconite," says he, "may be useful at the commencement of the disease, but when the effusion is pretty considerable, it becomes only an accessary element in the treatment, and it then becomes necessary to join with it purgatives, diuretics, and blisters."

Dr. T. has used the preparations of aconite in typhus, and in inflammatory fevers, but without success. He is induced to believe that aconite would be a useful remedy in syphilitic eruptions ; but having never administered it in these diseases he is unable to bring any facts to the support of his opinion.

He cannot say anything of the value of this medicine in chronic psoric affections.

Dr. T. has had a great desire to try the administration of aconite, in accordance with the advice of M. Tessier (of Paris), in the purulent diathesis. He would state, however, that the purulent diathesis is a disease so terrible, and which carries of patients so rapidly, that he would hardly dare to trust to the action of aconite, except as an adjuvant, to arrest its progress.

Besides the diseases already enumerated, Dr. T. thinks that there are many, in the therapeutics of which aconite may enter as an accessory. As a general rule, he observes, we may derive benefit from its employment whenever we are called upon to treat a disease, in which the cutaneous action is abnormally performed.

*Mode of Administration.*—In rheumatism, neuralgia, or in any other painful disease, where the anodyne effect of aconite is desired, Dr. T. gives from 10 to 20 drops of the tincture, increasing the dose gradually to 3, 4, 5, and even 8 grammes a-day. In courbature, or catarrhal fever, he gives only from 5 to 10 drops of the tincture in the 24 hours: by this means he reduces the pulse, and lowers all other febrile symptoms, without producing any of the phenomena of intoxication. He recommends that the medicine be taken some time either before or after a meal. "Of course," says he, "it must be used with more reserve in the cases of children, than in those of adults."

Dr. T. prefers the alcoholic tincture of aconite to the other preparations of this medicine, considering it as being more reliable than the extract. The dose of the extract, he says, is from 6 to 10 centigrammes, or even to 1 gramme a-day.

We give here the recapitulation of Dr. T's conclusions.

1. Aconite is a very useful medicine, and is applicable to a great number of diseases.

2. It is a narcotic less active than opium, belladonna, and the datura, but which nevertheless may be of great service in painful diseases, especially in those which recognize for their cause a serous, catarrhal, or rheumatic fluxion. It is less successful in the treatment of pains proceeding from diseases decidedly inflammatory.

3. The characteristic property of aconite is, to act on the functions of the skin. It exerts a special eliminatory influence on this membrane, that renders it useful, either as a principal or adjuvant remedy in all diseases where disorder of the cutaneous action constitutes a prominent part, especially in courbature, catarrhal fever, grippe, angina, and acute pulmonary catarrh, articular and muscular rheumatism, especially those cases attended with but little fever, and slightly marked inflammatory symptoms, rheumatic neuralgia, etc.; and in all affections where a morbific principle is retained in the meshes of the cutaneous tissue, and alters its normal organization, as in all the exanthemata (rubeola, variola, scarlatina, miliaria, urticaria, erysipelas, etc.)

4. Aconite is never directly antiphlogistic. It cannot replace bloodletting. It simply diminishes, in many diseases, the frequency of the pulse by quieting the pains which occasioned the fever, or by facilitating the elimination of the morbid principles which kept it up, as in erysipelas and measles.—*Southern Medical & Surgical Journal.*

